# Developing a Scientific Virtue-Based Approach to Science Ethics Training

**DOI:** 10.1007/s11948-016-9757-2

**Published:** 2016-01-27

**Authors:** Robert T. Pennock, Michael O’Rourke

**Affiliations:** 1 Lyman Briggs College and Departments of Philosophy and Computer Science & Engineering, Michigan State University, 919 E. Shaw Lane, Rm. E35, East Lansing, MI 48825-3804 USA; 2 Department of Philosophy and AgBioResearch, Michigan State University, 536 South Kedzie Hall, East Lansing, MI 48824-1032 USA

**Keywords:** Responsible conduct of research, Science ethics, Scientific virtues

## Abstract

Responsible conduct of research training typically includes only a subset of the issues that ought to be included in science ethics and sometimes makes ethics appear to be a set of externally imposed rules rather than something intrinsic to scientific practice. A new approach to science ethics training based upon Pennock’s notion of the scientific virtues may help avoid such problems. This paper motivates and describes three implementations—theory-centered, exemplar-centered, and concept-centered—that we have developed in courses and workshops to introduce students to this scientific virtue-based approach.

## Introduction

Science, like other well-established cultural practices, has an inherent normative structure—a set of values, both epistemic and ethical, that guide and govern its practitioners (Douglas 2009). Responsible conduct of research (RCR) training, whether done in workshops or courses, covers only some of these values. For historical and practical reasons, RCR training has tended to focus on a variety of standard topics, including protection of human and animal subjects; data fraud, fabrication and other issues of research integrity; authorship, credit assignment and other issues involving researcher relationships; conflict of interest and other issues of institutional integrity; social responsibility and the like (Pimple 2002). Traditionally, RCR training involves laying out rules and professional expectations in these areas, often explaining these in terms of egregious cases of misconduct that sensitized the profession to the need for explicit rules, and typically justifying them philosophically in terms of rights and duties or utility (CITI 2015). As part of this training, students are introduced to various legal or quasi-legal requirements and procedures a researcher must comply with, including federal regulations, due process, penalties for infractions, and the roles of the Research Integrity Officer and Institutional Review Boards (IRBs) (cf. Steneck 2007). Explanation of such rules and procedures is often supplemented by case studies, real or hypothetical, that illustrate examples of misconduct that should be avoided, confronted, or resolved (cf. NAS 2009). RCR is eclectic to be sure, but we will argue that there remain other topics that deserve to be taught under a broader heading of *science ethics*. Moreover, we have observed some common difficulties when RCR training is done as described above.

One problem is that it can give the false impression that science ethics is just a matter of knowing and following a given set of rules. Worse still, when couched in a compliance-based framework, RCR can appear reducible to a checklist of rules that one simply must tick off. Such a view is simplistic, of course, for difficult cases require ethical judgment, understood as the outcome of principled deliberation among complex alternatives in which trade-offs involving competing values are carefully weighed. Using real or hypothetical cases about ethical dilemmas can help students better appreciate the complexities of rule-based choice, but these can often swing them to the opposite extreme, making them think that ethics is relative or unresolvable (Wolpe 2006). In a full course, instructors have the time to help students make their way through ethical relativism and develop analytical skills for adjudicating among prima facie rules (McGuffin 2008), but one-off RCR training workshops mostly have to gloss over these difficulties.

A less obvious, but more serious issue is that, with its focus on compliance and rule-following, traditional RCR comes across as legalistic (Pennock 2015a). There are several problematic effects of framing RCR in this way. One all-too-common effect of a legalistic model is that it makes RCR appear to be a burdensome bureaucracy. Researchers too often come to view compliance-focused rules and regulations as just more red tape that gets in the way of science. This can give rise to a cynical attitude toward the IRB, for example, that its forms are annoying hoops to jump through or to avoid if possible. A second, equally problematic effect is that RCR may begin to feel to scientists like a police state; it not only emphasizes rules and compliance, but also enforcement, procedures, and punishment. Researchers come to see themselves as under scrutiny by the ethics police. The Research Integrity Officer (even the name makes it sound like they will be in uniform) is someone who enters the scene when possible rule-infraction or misconduct needs to be investigated and prosecuted. Framed in this way, scientists who break the rules are criminals.

Put more generally, the legalistic model makes RCR seem like something that is *imposed* upon science *from without*. The unfortunate result of traditional compliance-based RCR training is that ethics too often become seen as an interference with research or at best a necessary burden. It fails to motivate scientists to think about their research in ethically responsible ways except perhaps as a defensive measure. This is hardly a positive way to create an ethical culture.

What can we do to change this attitude? How can we teach RCR and science ethics generally to make these problematic effects less likely to occur? We want science students, as well as working scientists, to see values as part of the fabric of science itself. Responsible conduct should not be viewed as something foreign, imposed from without, but rather as something native and familiar, arising out of science’s goals, methods and practices. In this paper we present one way to do this based upon Pennock’s notion of *scientific virtue* (*SV*).

This is a hybrid notion that is neither straight virtue ethics nor philosophy of science, though it draws upon both. Virtue ethicists ask about what character virtues are conducive to being a flourishing human being, but the scientific virtues are those traits that make for an exemplary scientific researcher. Philosophers of science have gone into great depth about the qualities that make for better scientific theories, but the SV perspective looks also at the qualities—especially the character traits—that make for better *scientists*.^1^ This is not a descriptive thesis about the actual traits of all or even most scientists, but rather a normative thesis about scientific aspirations and ideals—it is about the *exemplary* scientist. Scientists are exemplary to the extent that they embody the virtues that dispose them towards the ideal practice of science’s distinctive methods for achieving its goals. By focusing on the character of the exemplary scientist in this normative sense, the SV approach embraces both epistemic and ethical values, connecting them in a way that links the nature of science to its responsible conduct (cf. Schienke et al. 2011).

The practical import of such ideals of scientific virtue has previously been shown to play out both broadly, regarding requirements such as the general responsibility to defend the integrity of scientific methods (Pennock 2006), and specifically, regarding issues ranging from just authorship attribution (Pennock 1996), to responsible research funding and conflict of interest (Pennock 2002), to approaches to dealing with socially controversial subjects such as human cloning (Pennock 2001). Later in this paper we’ll note some additional connections to core RCR topics such as fraud and fabrication, and to issues in science ethics that go beyond traditional RCR, such as responsibilities scientists have with regard to broader social issues, including biases based on gender, race and so on. The SV approach also highlights the significance of epistemic values in science such as the importance of attentiveness and the meticulous collection and analysis of data (cf. Steel 2011).

However, this is not the place to review or delve into philosophical arguments about these in detail. We intend these initial considerations to motivate interest in such an alternative, virtue-based approach to RCR and science ethics, and we will henceforth proceed to describe how one might pursue such an approach. Thus, this paper focuses on educational training, specifically on ways to integrate the scientific virtues into RCR and science ethics. Drawing on the experience of our own experiments in developing courses and workshops using this approach, this paper will describe three ways to do this—theory-centered, exemplar-centered, and concept-centered—and discuss the relative merits of each. The concluding section gives an informal general assessment of the advantages of an SV-based approach and a brief discussion of a pilot assessment study that is currently underway.

## Theory-Centered SV Approach

From a philosopher’s point of view, it is natural to begin developing a SV-based approach to RCR and science ethics in light of ethical theory. Moreover, professional ethics courses, whether taught by philosophers or not, are typically organized around a systematized theoretical framework. Classic textbooks in medical and engineering ethics, for instance, mostly follow this approach (Beauchamp and Childress 2012; Whitbeck 2011). If an instructor aims to convey the full logic and justification behind RCR and science ethics, this is clearly an excellent approach. Because RCR and science ethics have not generally been conceptualized in a virtue-theoretic framework, a theory-centered SV approach must first provide a general introduction to virtue ethics, starting with its roots in Aristotle.

According to Aristotle, a character virtue is a habituated tendency or inclination—“a settled disposition of the mind” (Aristotle 1889)—to have appropriate feelings, which in turn leads one to act in appropriate ways.^2^ Specifically, the virtues of a human being are those that accord with human purpose and function and so promote human flourishing. Happiness in its robust Aristotelian sense is not just a mental state but also an activity—one must exercise one’s distinctive human faculties to actualize them. In Aristotle’s view, the actualization of potential is judged in terms of a thing’s purpose (*telos*). Put in this way, virtues are what enable a person to function in a manner that will best achieve the distinctive and highest human aims.

Virtue ethics emphasizes that it is not enough to know what one should do; one must also *care* to do it. Possessing developed virtues means that one will feel emotionally *motivated* to act in the right ways. Justice, courage, prudence, and wisdom are among the virtues that Aristotle recommends as central to human flourishing. He also provides a framework for analyzing virtue, noting that it is a balanced condition, being neither excessive nor deficient in the requisite trait; moderation is the key. Virtue cannot be acquired just by learning rules, but must be developed by practice and by following the example of individuals who have already developed the practical wisdom (*phronêsis*) about how to achieve such a harmonious balance.^3^

### Pennock’s Scientific Virtues Account

Virtue ethics, of course, focuses on what it is to be a virtuous person, whereas our more circumscribed interest is in the professional virtues, here on those of the scientist. Thus, in a theory-centered SV approach to RCR and science ethics, instead of beginning with human nature, one starts with the nature of science. Science is a practice with its own goals and standards of excellence. The Philosophy of Science works to explicate these standards, including notions of scientific explanation, confirmation, methodology, and so on. An SV approach starts with a philosophy of science but goes on to develop a philosophy of the scientist, looking at what may be thought of as the scientific mindset or as scientific habits of mind. Scientific virtues are those character traits—what we may think of in this context as *practiced dispositions* which have a general biological basis, but which are given specific normative content by, and must be learned through, scientific practice—that are necessary for or conducive to achieving the aims of science. One may analyze the goals of science in a more or less fine-grained manner, but its central aim is to discover empirical truths about the natural world. The purpose of the scientist is thus, at least on a first pass, much narrower than the general purpose of a human being. There are a variety of traits that make one a better person, but this basic scientific goal helps us focus on the distinctive traits that a scientist should cultivate; because of science’s special aims, *curiosity* and *intellectual honesty* are the primary scientific virtues on this account. Other virtues play important related roles.

For example, science’s methods involve empirical testability; hypotheses should not be accepted merely on the basis of authority or personal preference, but must be tested and confirmed in terms of observational evidence that is in principle public and repeatable. Thus *skepticism* and *objectivity* are critical virtues. Repeatable empirical testing is not easy, especially when one must quantify results, so *perseverance* and *meticulousness* are also valuable qualities for scientists. One may take an analytic approach to elucidate these and other scientific virtues, and one may also find them exemplified in narratives or embodied in exemplars. We can go on, but this is not the place to flesh out and defend a full list of scientific virtues. Pennock’s account is built on a combination of philosophical reasoning and historical research, supplemented by informal interviews with many scientists over the last 15 years. His Scientific Virtues Project <www.msu.edu/svp> is now investigating this further in a systematic national survey of scientists.

We do not claim that presenting a set of scientific virtues will be sufficient in and of itself to produce ethical behavior in science. Even in a theory-based course one cannot side-step the practical and political complexities that researchers must confront in messy real world circumstances as well as external pressures that can threaten even core scientific values. Ethical treatment of human subjects, animal welfare, and other such issues in science and ethics will still require special attention (Rollins 2006). A full ethical treatment must also cover how traits can tilt from virtue to vice if taken to an extreme. A virtue-based approach does not assume that scientists are saints—*humility to evidence*, for instance, does not preclude arrogance or other forms of prideful behavior (Pennock 2015b)—and it will take thoughtful intention to develop the necessary practiced dispositions. Nevertheless, we argue that an approach that highlights how exemplary character traits that arise from the goals and methods of science can help overcome some common problems. As David Hume pointed out, one may know rationally what one should do but simply not care to do it (1983, Bk. II, Pt III §iii). Traditional rule-based ethics always has this problem when considered in isolation. In a virtue-based framework, on the other hand, the interconnections between the aims of a practice and the character and motivation of someone pursuing that practice help alleviate this problem. It can be helpful to think of the logical structure here as a kind of hypothetical imperative—if one wants to achieve aim A, then do behavior B. If one wants to do B well, be a C (i.e., have character C).^4^ Having the kind of character traits that will incline one to achieve scientific aims means that one is already motivated to behave in appropriate ways.

With an SV framework in place, standard RCR topics may be presented in a fresh light. To give just one example, consider the issue of data fabrication. A traditional RCR workshop might present the rules against data fraud and fabrication in professional codes of ethics, discuss cases when scientists violated these rules, and explain the procedures for investigating suspected cases and the penalties for violations, from paper retraction to job termination. Such information is useful, and highlighting legal deterrents might scare some people into thinking twice before fabricating data, but the SV approach is better than this kind of legalistic approach if one’s aim is to cultivate a true culture of integrity. On an SV approach, the issue of data fabrication could arise naturally as an outgrowth of a discussion about virtues such as objectivity and intellectual honesty, but students get the point most directly when they consider it in relation to the core scientific virtue of curiosity. The curious scientist wants to discover something or find the answer to a question or test whether some hypothesis is true—in short they want to know something about the world. The very idea of fabricating data is inimical to this basic scientific attitude. It is not that one shouldn’t fabricate data because you might get caught and punished for violating a rule, but rather that the very idea of fabricating data violates what it means to be a scientist. In this kind of way, an SV-based account of RCR reveals behavioral implications that come from within the practice of science.

### Difficulties

A theory-centered approach allows one to be systematic and to elucidate the logical structure of RCR in science, but we should note several practical difficulties for doing RCR training in this way. One temporary problem is that, because a virtue-based approach to RCR training is still very new, there are almost no standard classroom materials. Pennock is completing a book on the topic, but otherwise few materials are currently available.

A more general problem is that science students and faculty typically lack a background in philosophical theory. What this means is that theory-centered RCR courses have to start from scratch and provide an introduction to ethics just to get things going. However, many science students lack that level of interest, and if the faculty are not philosophers, they may lack the expertise necessary to teach the material. Just as non-science majors opt for science courses that are taught without the mathematics, so non-philosophy majors often want a course that sidesteps the logical and theoretical foundations and complexities. Even though philosophers would argue that theory must be primary, at least in a justificatory sense, we must recognize that it is not appropriate for every audience and is not always the most effective means of training.

While theory provides frameworks that students can use to organize the concepts and issues that constitute RCR, it can seem rather abstract to those who are not philosophically inclined. Approaching RCR in terms of cases first and then bringing in theory to help resolve dilemmas is one good approach, but an SV-based approach also allows another alternative—one that is centered on exemplary persons who embody the relevant virtues. We now turn to a description of that approach.

## Exemplar-Centered SV Approach

Virtue theory holds that one becomes a virtuous person in part by learning from and modeling oneself after individuals who themselves exemplify human virtues; someone who embodies the traits that make for human flourishing can serve as an exemplar of their operation and effect. Acquiring the traits that make for exemplary science is much the same. Although role modeling is probably best done in personal mentoring relationships (Bird 2001), it can be approximated in the classroom by what may be thought of as a virtual apprenticeship with exemplary scientists.

Pennock has presented elements of such an exemplar-centered approach in introductory courses, but finds it to be especially effective in upper-division classes after students have already completed a variety of science courses.^5^ Here we will describe implementations in senior seminars at Michigan State University in Lyman Briggs College, MSU’s special residential program for the study of science and society. Most Briggs students major in science and go on to graduate or professional school in science or medicine.

### Course Structure and Rationale

As in a theory-centered approach, the goal this course is to have students explore science ethics and the scientific mindset, especially the character virtues of the exemplary scientist that they should try to emulate, and how these relate to traditional RCR topics. The difference is that an exemplar-centered course is organized around consideration of exemplary scientists, carefully selected to allow students to explore the scientific virtues from different points of view in a wide range of contexts.

To allow comparisons across scientific disciplines, it works well to include scientists from fields ranging from physics and biology to computer science. It is also revealing to compare and contrast science to other professions, such as engineering and medicine, which emphasize different virtues (e.g., innovation or compassion) because of their different aims and methods. Such a disciplinary range also helps ensure that students have role models from within their own major fields.

In selecting exemplary scientists, it is valuable to pair historical and more contemporary scientists in a field. There is much to be learned from scientific giants like Charles Darwin and Albert Einstein, but including less well-known figures such as Barbara McClintock and Richard Feynman helps students see how the scientific virtues are broadly exemplified. Historical sources reveal the roots of the scientific culture, especially in the Scientific Revolution where these values are articulated most self-consciously because natural philosophy is seen as a new movement. It works especially well to start with Benjamin Franklin’s autobiography (1916), not only for his historical significance, but also because Franklin explicitly wrote about virtues, their significance, and how he tried to develop and fortify them in himself. As a pioneering scientist, Franklin serves as a model himself of exemplary character traits but he is unusual in also theorizing about the general development of virtues explicitly and systematically.

As with any culture, science mostly takes its own cultural values for granted; even those who are aware of those values rarely have occasion to talk about them directly. Indeed, Franklin’s discussion, like Aristotle’s, is about the virtues of a human being rather than those of the scientist, but it provides a useful introduction to thinking in this way, and one may then ask students to look more closely at Franklin’s (and other scientists’) work to try to discern what specifically scientific virtues might be teased out. Having students do this as an inquiry-based exercise gets them actively involved in thinking about virtues and how they are expressed, and is another advantage of this exemplar-centered approach. The idea is to encourage students to survey the contours of the scientific character on their own by triangulating from different source materials. An exemplar-centered approach allows a wide range of materials beyond the usual textbooks and articles, which as noted above still remain few and far between in this area. On this model, the primary texts are biographies and autobiographies of exemplary scientists, but may also include eulogies, obituaries, commencement addresses, documentaries, and even fictional depictions of scientists.

Both biographies and autobiographies have their own advantages. For Charles Darwin, for instance, one has a wealth of biographies to choose from. Nothing matches Janet Browne’s magisterial biography (1996, 2003) to give the fullest picture of Darwin’s life, work, and times, and Desmond and Moore’s nuanced biography (1992) is another excellent choice. In practice, however, students are more engaged and get a better sense of Darwin’s character from reading his *Autobiography* (1958). Although autobiographies provide less historical context and miss the measured judgment of a third person account, this is made up for by the immediacy of first person narrative, which can be more important for our purposes. One way that students absorb virtues is when the intellectual is linked to the emotional. Autobiographies at their best are personal and even intimate—both Franklin and Darwin were writing primarily for their family—so a reader can *feel* the character of the writer. They make it easier for readers to identify with the scientist. It allows them to think “I could be like that”, which is a key intellectual step in becoming virtuous, followed closely by “I *want* to be like that,” which is the critical motivational step.

Richard Feynman’s autobiographical books are also excellent (1985, 1988) for just this reason. That Feynman was a genius and Nobel laureate gave him license to be eccentric, but the overwhelming perception one gets is of someone who just lived and breathed science and who could not help but share that passion. Feynman is an engaging and likable character who personifies scientific curiosity. Even better than the books are the filmed interviews with Feynman, which have been broadcast in various forms over the years (BBC/PBS 1981). Indeed, these interviews have such a high value from an SV perspective that they should be at the core of any exemplar-centered SV course. Feynman is the modern epitome of the scientist role model. He is a rare case of a scientist who not only embodied the core scientific virtues, but also had thought about them explicitly and deeply and could articulate them both directly and through anecdotes. He was a scientific storyteller and saw himself as such. It is also easy to use Feynman’s discussions, for instance about honesty in science or about the causes of the Challenger disaster, to highlight how scientific virtues can help avoid some common RCR problems.

Whether one uses biographies, autobiographies or some other source material, it pays to be pedagogically transparent about the process of triangulation one expects students to do. Each time one introduces a new kind of source material it is useful to devote up to half a period to consideration of its value and limitations, and how it fits with an SV approach and illuminates our understanding of community norms. There are a variety of excellent documentaries and docudramas about important scientists, for instance, which can occasion fruitful discussion about how scientists’ character traits are portrayed in each.^6^ Virtue theory holds that character is akin to a dramatic role, and that “stock characters” in plays often are the way that particular virtues are displayed (MacIntyre 1981, pp. 27–31), so docudramas can sometimes be as revealing as documentaries once students are given the theoretical framework to understand how to analyze them. Looking at the same scientist through different source materials gives students a much richer appreciation of their character.

An exemplar-centered SV approach permits an eclectic range of modes and methods. Because learning is especially effective when students can uncover and explore the scientific virtues on their own through the lives of exemplary scientists, student-guided discussion works well, punctuated by short lectures that introduce philosophical concepts and theory as they become salient. Virtue theory holds that one learns to embody the virtues in part by practice and habituation, so the challenge is to structure the class accordingly. Instructors can encourage this in a variety of ways, such as by asking students to intentionally practice one or other scientific virtue for a day and then report upon the experience. It works well to have them write daily blogs to reflect on their reading and discussion, and then have them work in pairs to digest and present their understanding of the readings. Another novel approach is to give students the option of putting on a dramatic performance as an alternative to a formal class presentation. Not every student is equally at home with such role-playing scenarios, but those who are play their parts with relish and the exercise gives them a chance to try the characters on for size. One ambitious group of students dramatized scenes from the life of Ada Lovelace. Another did skits drawn from a novel about a scientist and followed it up, still in character, with a full class discussion about some of the elements of the piece with the rest of the class being asked to play along as though members of a debating society that had been depicted.

As these novel-based skits illustrate, an exemplar-centered SV approach can fruitfully draw from fictional as well as historical sources. Because our interest is in the normative structure of science and character ideals, fictional narratives—plays, novels, films and so on—can often be as informative as non-fiction. Brecht’s *Life of Galileo* (2015), Lewis’s *Arrowsmith* (1925), and even Sagan’s *Contact* in book (1985) or movie (Zemeckis 1997) form, can be shapers of scientific community norms in part because the fictional form allows character traits to be exaggerated for effect or exemplified in contexts that highlight their significance.

In addition, a few more unusual kinds of source materials turn out to be useful in an exemplar-centered SV approach. As noted above, the deepest values of a culture are often unarticulated precisely because they are taken for granted—one doesn’t talk about them; one just lives them. However, cultures typically have special *occasions* when it is deemed appropriate to speak directly about these deep values and one may profitably look there to find them articulated. Not surprisingly, these regularly occur as one enters or leaves some important life or professional stage, and they often involve public addresses of some sort, because they are occasions whose point is in part to affirm the values of the community involved. In disciplinary contexts, these may take the form of initiation ceremonies of some sort, as well as award speeches or memorial services. Phi Beta Kappa initiations always include a “charge to initiates” which admonishes them to follow the ideals of companionship and zealous research. The initiation ceremony for Fellows of the American Association for the Advancement of Science always includes a speech from a notable scientist who talks about their research career. But unlike a talk at a professional conference where one simply presents one’s data and findings, these are occasions where the scientist typically tells the story of their career and reflects upon setbacks, highlights, collaborations, and lessons learned along the way. University graduation/commencement ceremonies participate in both, with the completion of one’s college training and the beginning of one’s post-graduate career, and here too the expectation is that the guest speaker will speak to the ideals that graduates have learned and are expected to exemplify going forward. It is easy to be cynical about such speeches, filled as they often are with clichés and platitudes, but it would be a mistake to dismiss them. In part because they speak to what everyone is already expected to know, addresses at such occasions can provide a rich source of information about the values that a community holds to be important and constitutive, in ways that are broader than RCR training typically covers.

Probably the most significant occasion is at the end of life, as this is the point at which individuals are presented in their best light; their qualities are named and their life and character is celebrated. For this reason, obituaries and eulogies are also interesting materials to examine. Although the practice has become less common in recent years, in the past scientific journals regularly published scientists’ obituaries, sometimes quite lengthy ones, that went beyond a summary of their research and also spoke of their scientific lives and character. It works well, for example, to have students to read one or more of the long obituaries that were published on Darwin’s death and then have them find the obituary of some other scientist they are curious about. Having students compare these makes for a lively discussion about what is or isn’t highlighted by the scientific community as it reflects on the lives of departed scientists. Again, for our purposes, it does not matter whether such accounts are completely accurate from a descriptive point of view—perhaps the scientist did not quite live up to the ideals as presented. For our normative investigation we do not care so much about that as what those ideals are thought to be. A discussion of scientific obituaries also provides an opportunity to talk about how to judge success or failure in virtue terms. Solon said that one cannot judge whether a person is truly happy until they are dead and one can see the full sweep of their lives. This long-term perspective provides a useful vantage point from which to think about broader notions of scientific integrity.

Another area that goes beyond traditional RCR topics is how scientists should deal with broader social issues, such as religion, gender, sexual orientation, class, and race. Our general heading of science ethics also makes room for consideration of scientists’ social responsibilities and other topics that relate to what the National Science Foundation calls the “broader impacts” of scientific research. An advantage of the exemplar-centered approach is that it allows such issues to be examined concretely rather than abstractly, through the experiences of real individuals. In this way, to give just one example, social prejudices may be seen as objectively real and also may be judged with more subtlety rather than simply in terms of stereotypes.

As the son of a prominent physician, Darwin’s social position provided him with important advantages for someone who was proposing such a revolutionary view, but other scientists had to overcome class barriers. Michael Faraday, a bookbinder whose scientific mindset led him to the highest levels of scientific achievement and recognition in his period, is a useful exemplar for examining these issues. For a closer comparison, one could refer to Alfred Russel Wallace, who needed to sell exotic beetles to collectors to help to fund his research. Darwin’s class and connections also helped buffer him from the religious fallout of his discovery (Desmond and Moore 1992), but the conflicts between scientific and religious values and virtues are not easily overcome. One could examine many of these issues using Galileo as the exemplar. Moving to the 20th century allows one to look at more recent examples where scientific values met religious and other social challenges. Einstein was only the most famous of physicists in his time who faced anti-Semitism and had his “Jewish science” dismissed out of hand. Lise Meitner’s scientific research was abruptly interrupted because of such prejudice.

Meitner’s life also serves as a way to explore scientific virtue and gender issues, as does that of Marie Curie, whose two Nobel prizes put her in the most rarified of scientific company, but there are plenty of other female scientists who could also serve this purpose. Barbara McClintock is a particularly useful exemplar for such discussions as she clearly articulated how her virtues as a scientist ought to dominate any biases she faced because of her gender (Keller 1983).

Alan Turing works well as an exemplar to highlight pioneering work in computer science. Sitting as he does at the border between basic and applied science, Turing’s scientific life provides a way to examine the different goals and thus different virtues of a scientific versus an engineering perspective. He also serves as another point of reference in considering scientific values in the broader societal landscape; Turing’s science was only a temporary refuge against the social prejudice he faced as a gay man. Similar issues have arisen for scientists who have had to deal with racism, and an exemplar-centered SV approach allows students to think about interpersonal and institutional biases that might hinder scientists who are members of under-represented groups and thereby hinder the progress of science.

Such cases show the value of an expanded notion of science ethics that goes beyond traditional RCR topics and incorporates a scientific virtue-based perspective. Especially illuminating is how such cases highlight common scientific values, such as truth-seeking and objectivity, that hold steady even in the face of different social challenges, and supply a useful antidote to philosophical views that discount these values. Power analysis as an explanation of dynamics in science tends to overlook and underappreciate basic scientific values such as these, which function as explanatory factors that are reflective of scientific practice more generally. Science is not exempt from the usual cultural prejudices, but such examples show how scientists have a value system based on curiosity and other distinctive virtues that provides a counter to those biases. In the end scientists do have a moral compass, based in their shared purposes, that should return them to the path of evidence and help them follow where it leads.

### Difficulties

An exemplar-centered approach works well when an instructor has both the luxury of time to allow students to slowly come to see the virtues through their own exploration of the lives of exemplary scientists and the expertise required to facilitate this exploration. Some majors require a professional ethics course, but others do not. In the latter case, even students with a deep interest in the subject may find it hard to fit a whole class in their schedule and seek to fulfill their RCR requirement in extra-curricular workshops. But time is at a premium in a workshop setting, and one must cut to the chase more quickly, especially with an audience of graduate students, postdocs, and faculty. For such a workshop setting, we now turn to a third approach that is centered around direct exploration of scientific virtue concepts.

## Concept-Centered SV Approach

Our hypothesis is that one can demonstrate how a good working understanding of the goals and methods of science implies an ethical structure. This is the sort of understanding that faculty typically have and graduate students and post-doctoral researchers in the sciences are acquiring. What this audience needs is an ethical vocabulary and a conceptual toolbox plus some thoughtful guidance to help them draw out these implications. Here we describe such a concept-centered approach that we have been developing and pilot-testing since 2011 at BEACON, an NSF Center for the Study of Evolution in Action at Michigan State University.

For this approach, we organize a dialogue-based workshop around particular scientific virtue concepts, each of which is the focus of a module comprising statements, or “prompts”, that are crafted to stimulate thoughtful discussion among the participants. Together, these modules constitute what we call the Scientific Virtues Toolbox (or SV Toolbox) instrument. This workshop approach is inspired by and modeled upon the structure of the Toolbox Project (O’Rourke and Crowley 2013). The original Toolbox instrument was conceived as way for interdisciplinary science teams to explore tacit assumptions about the epistemic and metaphysical foundations of scientific research (Eigenbrode et al. 2007). It includes prompts such as:

- Scientific research must be hypothesis driven.
- Validation of evidence requires replication.
- Objectivity implies an absence of values by the investigator.^7^

Participants are asked to indicate the degree to which they agree or disagree with each prompt using a standard Likert scale, which helps prime the dialogue.

The workshop dialogue structured by the original Toolbox instrument does not aim to teach a particular way to think about these issues; rather, it is a discovery mechanism that is intended to create a context within which a team can identify, examine, and negotiate among themselves the different assumptions they may have about scientific research. The SV Toolbox, on the other hand, does have content goals. Its prompts aim to guide participants towards a better understanding of the values that give structure to science and how these relate to RCR topics.^8^ For instance, the module on honesty in science includes prompts like the following:

- An honest scientist will not omit relevant data.
- Overselling the importance of a research project is as dishonest as fabricating data.
- Honest scientists are not required to act when they suspect another scientist of dishonesty.
- There are situations where one must be scientifically dishonest to do the right thing.

Each prompt articulates a relevant, normative perspective on the nature of intellectual honesty in science, and our goal in developing these is to cover a broad range of relevant issues while representing a number of different perspectives. We design some prompts (e.g., the fourth in this list) to be provocative, since their function is to generate discussion; further, we vary the valence of the prompts to encourage different reactions from prompt to prompt, a strategy that motivates participants to go slow and reflect on the prompts as they work through the instrument. We have been developing and pilot testing two modules per year and currently have sets on the purpose of science, curiosity, honesty, courage, perseverance, and humility to evidence, with others on the way.

### Workshop Structure and Rationale

The SV Toolbox prompts form the core of a workshop session that brings the scientific virtues concepts to the foreground and allows participants to explore their meaning, implications, and interconnections. The prompts are sometimes worded ambiguously so that participants have to disentangle different senses of terms on their own. As in the original Toolbox, participants are first asked to rate the degree to which they agree or disagree with each prompt on a standard 5-point Likert scale (ranging from strongly disagree to strongly agree). This primes the discussion by giving them a chance to first introspect. The scores also provide a way for participants to identify what may be unexpected patterns of similarity or difference in their initial opinions, which also makes for fruitful discussion.

The initial open-ended discussion of the prompts for a single module ideally lasts about 30 min and is lightly facilitated, allowing participants to work out and coordinate their own views on the issues. This is followed by more heavily facilitated discussion that can vary in focus with facilitator goals. Some important connections to bring out include how the scientific virtues arise out of science’s aims and methods so that participants come to appreciate the relationship between epistemology and ethics in science. Science is not just a way of knowing, but also necessarily a way of being. Facilitators should make it clear that these discussions are not focused on empirical claims about whether scientists *descriptively* have one or another virtue in greater or lesser degree than others. Rather, the focus is on *normative* questions about what we value in science—what we strive to be like and what we ought to do as scientists.

Often we find that key ideas, such as ways that scientific virtue relates to behavior, have already begun to emerge in the workshop dialogue, which provides a sufficient basis for subsequent guided discussion. In other cases, we take the general ideas that the prompts elicited and have the group explore their implications for action, specifically action that exhibits responsible research conduct. The curiosity module, for example, includes a prompt (“Fabricating data is compatible with scientific curiosity”) that often generates RCR-related dialogue in the workshop and can serve as a springboard for guided discussion of fraud and fabrication along the lines laid out above.^9^ We also encourage participants to consider ways that the scientific virtues function for scientists as individual researchers as well as for members of a research team and for the scientific community as a professional whole.

Additionally, we are beginning to make use of information from Pennock’s national survey. For instance, having workshop participants compare their own views to data from a representative sample of scientists helps them see whether their pre-reflective individual views coincide or diverge from the measured norms of the scientific community. We also can at this point present stories and anecdotes collected from the interviews with scientists. These are stories that researchers tell based on their own experience to illustrate the significance or the application of particular virtues in science. As noted previously, virtues are often conveyed and absorbed through narratives, so having a discussion around such stories exemplifies and reinforces their importance as community norms. They also often stimulate workshop participants to tell stories from their own experience, which provides another opportunity for reflective discussion.

Again, one advantage of this is that RCR and other issues of science ethics are seen as arising from within rather than being imposed from without. The SV Toolbox prompts are designed to stimulate participants to reflect upon science’s inherent aims and values beginning with their own understanding followed by its relation to the scientific community as a whole. We think that it is through this sort of information and through these kinds of interactions that an ethical culture is developed.

In the past 3 years we have conducted over two dozen workshop sessions, mostly for mixed groups of graduate students, post-doctoral researchers, and faculty, plus a few just with undergraduates. Typical group size is between 8 and 12 participants, but we have run groups as small as six to as large as fifty. For large workshops we have breakout groups of five or six participants for discussion of the SV Toolbox prompts, bracketed by whole-group instruction and discussion. Most of our workshop sessions last 90 min, which is sufficient for an introductory talk plus two modules back-to-back, but we have also done single modules in a standard 50-min class period.

Although three to five people tends to be an ideal size for small group discussions for most kinds of topics, we find that it works best to have slightly larger groups for SV Toolbox RCR discussions. Eight to ten seems to be ideal for a workshop group. Part of the purpose of SV RCR sessions is to create circumstances where community values can be teased out and then reinforced. If groups are too small, it is hard to recognize the patterns of values or see when a position is an outlier. There can of course be outlier views in any group—someone who thinks that lying on a grant proposal is acceptable so that one can fund one’s research, for instance—but these are more easily recognized as anomalous in a group of ten compared to a group of three.

For similar reasons, discussions are richer if one can include a mix of participants at different career stages in a group. Graduate students benefit from the practical wisdom of senior researchers, and faculty benefit from being reminded of the perspective of the novice and having the opportunity to be mentors and to pass along lessons they have learned. Of course, for these mutual benefits to arise naturally in discussions of the shared values and experiences of the scientific community, it is important that these be *balanced* discussions, with all participants feeling free to speak. The downside to a mix of faculty and graduate students is that the graduate students can remain silent and defer to the faculty; facilitators should be on guard for this possibility and work to ensure balanced participation from all members of the workshop.

We have experimented with different degrees of facilitator involvement in the SV Toolbox discussions themselves. In many cases we find that it works well to allow the participants to discuss the prompts in an open-ended manner in whatever order they wish with minimal interruption, which is the typical Toolbox facilitator approach (Looney et al. 2013). In addition to helping participants feel the sense of ownership that arises when people explore concepts in their own way, this also makes it much clearer that scientific values truly come from within the community rather than being imposed from without. However, depending upon the facilitator’s learning goals for the session and the makeup of the group, it is sometimes helpful to take a more active role even at this stage to guide the discussion—in much the same way that Socrates would be a “midwife” in a dialogue—to help key ideas emerge.

Finally, we recommend having workshops that are long enough to do two modules back to back. While there is certainly value even from focusing on a single virtue and its connection to some RCR topic, there is a greater benefit when participants can explore virtues in relation to each other. We intentionally construct modules so that prompts in one may link directly or indirectly to prompts in another. A major reason for this is that virtues have interconnections and are mutually supportive. This is related to the thesis, articulated variously by Socrates and Aristotle, of the “unity of virtue” (Plato 2009, Aristotle 1889). In one sense, this could mean that all virtues are actually just aspects of a single trait, but in another sense it could mean that the virtues are so tightly integrated that a person could not really have one without also having the others. Either way, the point is that virtues cannot fully be understood in isolation from one another. By having workshop participants consider at least two modules, they begin to recognize these interconnections. A second reason for pairing modules is that it starts to give participants practice with the balancing of virtues and the development of ethical judgment. For instance, perseverance is necessary to keep a research project going in the face of the usual experimental setbacks every researcher encounters, but a scientist must be ready to give up a line of research if the evidence accumulates against a hypothesis. Juxtaposing modules on perseverance and humility to evidence allows participants to explore how these values fit together.

### Difficulties

A concept-centered approach begins with smaller units of analysis than theory-centered or exemplar-centered approaches, and as a result lends itself to delivery in relatively brief but intensive workshops. This is useful for those who do not have the time to take a course in science ethics, but the concentrated nature of this experience has its downside. First, the brevity of the experience gives those who are new to the scientific virtues little time to reflect on what they are and how they relate to their lives as scientists. This can be offset to some extent by follow-up experiences, which is part of the approach as we have implemented it—those who pursue RCR training in BEACON will participate in at least one of these workshops per year, with subsequent workshops introducing them to new virtues as we indicated above. Second, there is a risk that a 90-min exposure to this approach will leave the connections between RCR concerns and the scientific virtues underdeveloped. In general, of course, the challenge for those engaged in RCR education is to enable students to take what they learn and have that shape their actions in ethically complex situations. To help address this difficulty, we have included more heavily facilitated, guided discussion designed to connect the dots between individual insights about the scientific virtues and responsible research conduct. We do acknowledge, though, that designing and delivering robust follow-up experiences will be more difficult for those who lack familiarity with the scientific virtues.

## Conclusion

We have described three ways to implement a virtue-based approach to RCR and science ethics, centered in theory, exemplars, and concepts. Of course, these three approaches do not exhaust the possibilities. Approaches centered on case-based modules or role-playing scenarios, for instance, are other options. Role-playing is not necessarily a comfortable mode for introverted scientists, but if that resistance can be overcome, it does have the advantage of fitting with the idea that virtues are passed on through narratives and are made visible through their embodiment in characters. Such possibilities deserve to be developed and investigated. For the moment we will continue to focus on developing curricular materials and assessing the three models we have described in this paper. Each has its own advantages, depending upon the audience and circumstances.

In addition to its use for the science students on which we have focused, a theory-centered SV approach can be valuable for philosophy majors and other students who want to delve into the logic and philosophical justification of the scientific virtues and how they are connected to both epistemic and ethical values in science. For science majors and others who are not inclined to get into the details of philosophy of science and ethical theory, an exemplar-centered approach provides a novel entrance into the scientific character virtues through an exploration of how they are embodied in the lives of exemplary scientists. We do not mean to suggest that one can or should dispense with theory in an exemplar-centered approach. Theory is still useful, of course, but it is brought in only after students have first begun to explore the ideas on their own through the lives of these scientists. Finally, we discussed a concept-centered approach that works especially well for intensive RCR workshops to train graduate students.

All three of these SV-based approaches appear to avoid some of the difficulties that we noted about traditional rule-based RCR training. One major benefit is that the scientific virtues are seen as arising from within science with philosophy helping to organize and explicate them, so science ethics becomes recognized as part of what it means to do science, rather than as something imposed from without. We are now assessing this formally. Specifically, testing is underway to compare participant reception of this scientific virtues approach to traditional RCR training. We are also testing specific SV Toolbox modules, looking at pre-post workshop data to document whether and how participants’ views and attitudes change. We will report on these formal effectiveness studies in future papers.

Our goal here was to introduce the scientific virtue-based approach as an alternative that we can recommend as a promising new way to teach science ethics from the inside out. Informally, we can report a very positive response from participants. For example, in our pilot tests of the concept-centered approach, one representative participant had this to say in a post-workshop survey: “The (SV Toolbox) exercise was much more motivating than traditional RCR. It made me want to be a better scientist immediately.” If a scientific virtues-based approach can consistently foster this sort of attitude, it will be a worthy complement to other methods of science ethics training and help promote a culture of scientific integrity grounded in ideals that are part of the very fabric of science.

## Footnotes

1 Here we will mostly focus on traits scientists should have as individuals, but the notion also encompasses characteristics of scientists acting in a community, including labs, professional societies, and other scientific institutions.

2 More completely, Aristotle says that a moral virtue is “a settled disposition of the mind determining the choice of actions and emotions, consisting essentially in the observance of the mean relative to us, this being determined by principle that is, as the prudent man would determine it" (1889, 1106b36–07a2).

3 For classroom materials, excerpts from Aristotle are good as a primary source, but contemporary texts require less interpretation. Rosalind Hursthouse’s *On Virtue Ethics* (1999) and Julia Annas’s *Intelligent Virtue* (2011) are good options. Annas’s account has some advantage for a training course in that she focuses on virtue as a learnable skill. Alasdair MacIntyre’s classic *After Virtue* (1981), which was the key book that revived virtue theory in contemporary ethics, is especially useful.

4 Note that these are not deductive relationships, but pragmatic imperatives. In the order of causation, character virtues make appropriate behaviors more likely, which in turn increase the likelihood of achieving the given aims. The context of training does add a degree of complexity, in that one way to acquire character virtues is to practice them, making B appear to be prior to C. However, what one is typically doing in this case is copying the behavior of a role model, M, who already has the character trait, so C remains causally prior in the larger picture. Pennock’s forthcoming book explains how the notion of scientific virtues as practiced dispositions helps make the conceptual and pedagogically practical transition from heritable, evolved tendencies to cultivatable scientific habits, but this is not the place to lay out a detailed theoretical account.

5 By extension, we believe that an exemplar-centered approach to SV ethics would be equally if not more effective if employed in a class with graduate students and post-doctoral researchers, given that they typically have an even more well-developed appreciation for the nature of science and scientific practice.

6 To give just a few examples, the *Nova* video *Einstein’s Big Idea* (Johnstone 2005), which is based on David Bodanis’ book *E* *=* *mc2: A Biography of the World’s Most Famous Equation*, is especially good, and there are several excellent docudramas about Darwin, including *Darwin’s Dangerous Idea,* Show 1 of the PBS *Evolution* series (2001), and the *National Geographic* docudrama *Darwin’s Darkest Hour* (Bradshaw 2009).

7 For the complete instrument, see the online materials associated with Looney et al. (2013).

8 While there are differences in emphasis between the original Toolbox approach and the SV Toolbox approach, the two are similar in that they aim to enable workshop participants to enhance both self and mutual understanding, which can increase the cohesiveness and functionality of the group. We thank James Foster for pushing us on this point.

9 Note that the prompt is phrased in a way that conflicts with the relationship between curiosity and fabrication described above, illustrating the point we made above that some prompts are written with different valences to motivate different reactions.
